# Gene set analyses for interpreting microarray experiments on prokaryotic organisms

**DOI:** 10.1186/1471-2105-9-469

**Published:** 2008-11-05

**Authors:** Nathan L Tintle, Aaron A Best, Matthew DeJongh, Dirk Van Bruggen, Fred Heffron, Steffen Porwollik, Ronald C Taylor

**Affiliations:** 1 Department of Mathematics, Hope College, Holland, Michigan, USA; 2 Department of Biology, Hope College, Holland, Michigan, USA; 3 Department of Computer Science, Hope College, Holland, Michigan, USA; 4 Department of Molecular Microbiology and Immunology, Oregon Health and Science University, Portland, Oregon, USA; 5 Sidney Kimmel Cancer Center, San Diego, California, USA; 6 Computational Biology & Bioinformatics Group, Pacific Northwest National Laboratory, Richland, WA

## Abstract

**Background:**

Despite the widespread usage of DNA microarrays, questions remain about how best to interpret the wealth of gene-by-gene transcriptional levels that they measure. Recently, methods have been proposed which use biologically defined sets of genes in interpretation, instead of examining results gene-by-gene. Despite a serious limitation, a method based on Fisher's exact test remains one of the few plausible options for gene set analysis when an experiment has few replicates, as is typically the case for prokaryotes.

**Results:**

We extend five methods of gene set analysis from use on experiments with multiple replicates, for use on experiments with few replicates. We then use simulated and real data to compare these methods with each other and with the Fisher's exact test (FET) method. As a result of the simulation we find that a method named MAXMEAN-NR, maintains the nominal rate of false positive findings (type I error rate) while offering good statistical power and robustness to a variety of gene set distributions for set sizes of at least 10. Other methods (ABSSUM-NR or SUM-NR) are shown to be powerful for set sizes less than 10. Analysis of three sets of experimental data shows similar results. Furthermore, the MAXMEAN-NR method is shown to be able to detect biologically relevant sets as significant, when other methods (including FET) cannot. We also find that the popular GSEA-NR method performs poorly when compared to MAXMEAN-NR.

**Conclusion:**

MAXMEAN-NR is a method of gene set analysis for experiments with few replicates, as is common for prokaryotes. Results of simulation and real data analysis suggest that the MAXMEAN-NR method offers increased robustness and biological relevance of findings as compared to FET and other methods, while maintaining the nominal type I error rate.

## Background

DNA microarrays measuring gene expression continue to grow in popularity, furthering our understanding of the genetic operation of organisms spanning humans to prokaryotes. Questions remain, however, about how best to interpret the wealth of gene-by-gene transcriptional levels measured in microarrays. Over the past few years, many statistical methods of analyzing gene expression data in the context of gene sets have been proposed to simplify and increase the impartiality of gene expression data analysis. Gene set methods are designed to aid the investigator in making biological sense of gene expression data by viewing genes under study in the context of *a priori *identified, biologically relevant, gene sets. Gene sets are groups of genes with some common characteristic (e.g. function, physical location in the genome, etc.). The most common methods of gene set analysis either use Fisher's Exact Test (FET) [1] or the newer Gene Set Enrichment Analysis (GSEA) method [2,3]. While FET and GSEA are the most popular, many other methods have also been proposed, see for example [4-18], and/or reviews of methods provided in [7,19-21].

Fisher's Exact Test was among the first methods proposed which used gene sets in statistical analysis of microarray data. In order to use FET, the genes in the experiment must first be dichotomized by classifying each gene as "up/down-regulated" (differentially expressed) or "not regulated." One method of dichotomization identifies genes with absolute values of log-ratios of expression scores above a certain cutoff as "up/down-regulated" and those below the cutoff as not regulated; see, for example, Schwartz *et al*. [22]. Once the genes have been dichotomized, FET compares the proportion of up/down-regulated genes in the set of interest to the proportion of up/down-regulated genes not in the set of interest. FET then uses the hyper-geometric distribution to compute a p-value based on the difference in the two proportions. As recently noted by Allison *et al*. [19] and validated through simulation by Ben-Shaul, *et al*. [17], the dichotomization necessary to use FET yields a loss of information which translates directly into a loss of statistical power, making it more difficult to identify real differences in regulation as statistically significant. Despite this loss of power Fisher's exact test is still often used in gene set analyses [22,23].

Other methods, like GSEA [2,3], have attempted to improve on FET. These newer methods do not dichotomize, instead they use the full range of quantitative gene expression data available (i.e. the entire sorted list of log-ratios of gene expression values). We will call these methods *non-cutoff based methods*. In most cases, the newer gene-set analysis methods that are alternatives to FET have been developed for human data. As such they assume that there are multiple replicates (one microarray/chip for each person in an experiment) of the data in order to conduct permutation based inference. For example, in an experiment comparing humans with a disease phenotype to those without, the statistic of interest (e.g. GSEA) is computed. Then, subjects are randomly assigned a phenotype status many, many times and each time the statistic of interest (e.g. GSEA) is computed. The statistic computed using the true phenotypes is then compared to the distribution of statistics based on random phenotype assignment in order to estimate the p-value (the measure of statistical significance of the strength of association between phenotype and regulation of the gene set). This type of permutation-based inference has been termed *subject-sampling *[20].

While there are many experimental and analytic similarities in DNA microarray experiments across organisms, prokaryotic organisms require some unique experimental considerations [24]. In particular, DNA microarray experiments on prokaryotes typically have many fewer microarrays (chips) per experimental comparison. For this reason, subject-sampling (permuting across the phenotype) can be impossible, since a moderate number of microarrays are necessary in order to have a sufficient number of permutations to correctly estimate small p-values. So, despite recent criticisms of FET, many of the non-cutoff based methods are not directly applicable to prokaryotic experiments.

There are two web-based software tools focused on prokaryotes, available for conducting gene set analysis. The FIVA tool [25] uses FET and a variant of FET proposed by Breitling *et al*. [16] which finds the optimal cutoff for "significant" vs. "non-significant" genes for each gene set. The JProGo tool [26] implements FET as well as three other non-cutoff based methods that compare the association measures (e.g. log-ratios) of genes in the gene set of interest with the association measures of the genes outside of the gene set of interest. The three non-cutoff based methods are the t-test, the Kolmogorov-Smirnov [K-S] test, and the unpaired Wilcoxon (Mann-Whitney U) test.

The non-cutoff based methods implemented by JProGO, however, are less than optimal. Specifically, the t-test requires a normality assumption about the microarray data, and is most powerful when testing for a difference in mean log-ratios between genes in and out of the set, unlike other methods which test for changes in standard deviation as well. The Wilcoxon test has received little consideration in the literature for use in gene set analysis and so it is unclear how valid and useful this approach is. The K-S test was considered by Efron and Tibshirani [8] who proposed an alternative measure, the MAXMEAN statistic. When comparing K-S to MAXMEAN, MAXMEAN performed better than K-S in both simulation and real data analysis. Efron and Tibshirani also compared MAXMEAN to a weighted K-S test (the aforementioned GSEA statistic) with a similar result.

After demonstrating that MAXMEAN is more powerful than K-S/GSEA, Efron and Tibshirani [8] compare MAXMEAN to two other statistics (we call these SUM and ABSSUM in this work). Efron and Tibshirani argue that neither SUM nor ABSSUM is robust with regard to changes in both the standard deviation and the mean, while MAXMEAN is powerful to detect both types of changes. The arguments of Efron and Tibshirani are made in the context of experiments on which multiple replicates are available and, thus, subject-sampling is possible. These methods [GSEA, ABSSUM, SUM, and MAXMEAN] have not been evaluated in the context of microarray experiments for which there are few, if any, replicates available.

In this work we consider five popular non-cutoff based gene-set analytic techniques originally proposed using subject sampling, (permuting the phenotype and, thus, requiring multiple chips) for use on prokaryotic microarray experiments with few replicates. We first demonstrate how these five methods can be implemented on experiments where subject sampling is not possible. Then, we conduct a simulation study comparing the five non-cutoff based methods with each other and FET for their ability to maintain nominal α (type I error rate; a measure of false positives) while giving high statistical power (a measure of true positives) relative to the other methods. We then compare the non-cutoff based methods to each other and FET on real microarray data sets obtained from experiments on *Salmonella enterica *serovar Typhimurium (*S. typhimurium*) and *Escherichia coli *K-12 *(E. coli)*. Lastly, we consider the biological significance of the findings in light of the different methods used.

## Results

The five non-cutoff based gene set analysis methods presented here are GSEA-NR (NR for non-replicated), ABSSUM-NR, SUMSQ-NR, SUM-NR and MAXMEAN-NR. These methods are compared to the traditional FET method. The GSEA-NR procedure uses a weighted Kolmogorov-Smirnov test statistic to compare the distribution of log-ratios of expression values for genes in the set of interest to those for genes not in the set. The ABSSUM-NR method uses the sum of the absolute values of the log-ratios for genes in the set as the test statistic, the SUMSQ-NR method uses the sum of the squared log-ratios for genes in the set as the test statistic, and the SUM-NR method uses the sum of the log-ratios as the test statistic. Lastly, the MAXMEAN-NR method uses the maximum of either (1) the average of the negative log-ratios times the percentage of genes in the set that have negative log-ratios or (2) the average of the positive log-ratios times the percentage of genes in the set that have positive log-ratios. We have modified the original versions of these methods to compute a p-value by comparing the test statistic for the gene set of interest to test statistics from random sets (in our case 50,000 random sets) of the same size selected from the experiment of interest (a method termed *gene-sampling *[20]). FET is implemented with four different cutoffs (0.5, 1, 2, and 3 on the absolute value of the log-ratio scale) and p-values are computed based on a hyper-geometric distribution. While we have implemented GSEA-NR, SUMSQ-NR, SUM-NR, ABSSUM-NR and MAXMEAN-NR using explicit gene-sampling, we note that FET uses gene-sampling implicitly [see [20]].

### Simulation Results

We conducted a simulation study to investigate the power of the different gene set analysis methods. Simulated gene sets, based on random log-ratios of gene expression values, were generated by random sampling from both normal distributions and from mixtures of normal distributions. Modelling with normal distributions allows us to consider gene sets that have relatively similar log-ratios of expression values. Modelling with mixtures of normal distributions is more flexible, and allows us to consider gene sets that are composed of subsets of genes that have similar log-ratios of expression values. Mixtures of normal distributions can create many different shaped distributions reflective of the many different types of log-ratios of gene expression distributions for genes within a gene set. For each of 2236 different combinations of parameters (mean of the log-ratios of gene expression values, standard deviation of the log-ratios, gene set size and whether or not the gene set was from a mixture of normal distributions; more detail in *Methods: Simulation study*) we simulated 2000 gene sets. For example, if the parameters were mean = 1 and standard deviation = 0.5 for a gene set size of 10, ten random values (to be treated as log-ratios) were selected from a normal distribution with mean 1 and standard deviation 0.5 to create a gene set. This same process was then done 2000 times for each of the 2236 combinations of simulation parameters. Simulation settings were selected to reflect gene set expression values from 18 experimental comparisons made on *E. coli *and *S. typhimurium*. Thus, the results of the simulation can be generalized to other experiments to the extent that the 18 experimental comparisons selected are similar to other experimental comparisons. We now describe the results of these simulations.

### Null hypothesis simulation

The null hypothesis simulation (data from gene sets with mean log-ratios of expression at or close to zero and small standard deviations) demonstrates that both GSEA-NR and SUM-NR can give false positive results, while the other seven methods [FET 3, FET 2, FET 1, FET 1/2, ABSSUM-NR, SUMSQ-NR and MAXMEAN-NR] correctly maintain the type I error rate, though they may be overly conservative. When the goal is a type I error rate of 0.05 (nominal α = 0.05), Table 1 shows that when the sample size is at least 10 and the standard deviation is small, the simulated type I error rate (empirical α) for GSEA-NR can be very large (averaging between 0.29 and 0.45). GSEA-NR also has an inflated type I error rate when the nominal α is less than 0.05 (results not shown). The lack of control of the type I error rate by all methods considered here is a virtue of the explicit or implicit gene sampling model used to assess significance. For a more detailed explanation see [20].

**Table 1:** Average Type I error rate (empirical α) when nominal α is 0.05

Set Size	True mean log-ratio of genes in the set	Standard deviation of log-ratios of genes in the set	GSEA-NR	ABSSUM-NR	SUMSQ-NR	SUM-NR	MAX-MEAN-NR	FET 1/2	FET 1	FET 2	FET 3
2–5 genes	0	0.01–0.05	0.0005	0	0	0.056	0	0	0	0	0
		0.10–0.20	0.005	0.001	0.0006	0.058	0.002	0.0004	0	0	0
	± 0.05	0.01–0.05	0.02	0	0	0.056	0	0	0	0	0
		0.10–0.20	0.01	0.002	0.0008	0.06	0.003	0.0005	0	0	0
10 or more genes	0	0.01–0.05	0.29	0	0	0.13	0	0	0	0	0
		0.10–0.20	0.03	0.004	0	0.14	0.0005	0.0001	0	0	0
	± 0.05	0.01–0.05	0.45	0	0	0.15	0	0	0	0	0
		0.10–0.20	0.08	0.006	0	0.15	0.004	0.0002	0	0	0

### Alternative hypothesis simulation

In the following sections we compare the five non-cutoff based methods with each other, the different FET cutoffs with each other, and then the best of the non-cutoff and cutoff based methods with each other. All comparisons in the upcoming sections are made in cases where at least one of the methods being compared yielded power > 80%, but not all methods being compared have power of 100%, where power is the percent of time that sets are correctly identified as significantly regulated.

### Comparing the five non-cutoff based methods

#### GSEA-NR

When the mean log-ratios of expression values is less than or equal to 0.5, the standard deviation of log-ratios is small, and the set size is large, GSEA-NR tends to have the best power as compared to the other four methods. However, situations with small mean log-ratios (near 0), small standard deviation and large set sizes are exactly when GSEA-NR has an inflated type I error rate (see *Simulation Results: Null hypothesis simulation*). Thus, while GSEA-NR is sensitive at detecting small and consistent changes in large gene sets, GSEA-NR appears to be overly sensitive and thus is eliminated from further comparative analyses.

#### SUMSQ-NR

SUMSQ-NR rarely (1% of the time) yields optimal power when compared to ABSSUM-NR, SUM-NR and MAXMEAN-NR. Further, in the few cases where SUMSQ-NR is optimal, ABSSUM-NR often (more than 95% of the time) yields power within 5% of SUMSQ-NR. Since SUMSQ-NR compares poorly to ABSSUM-NR, SUM-NR and MAXMEAN-NR we eliminate it from further consideration.

#### ABSSUM-NR, SUM-NR, and MAXMEAN-NR

Of the remaining three quantitative methods, we find that MAXMEAN-NR is the most robust to different distributions of log-ratios when gene set sizes are at least 10. Table 2 illustrates this by providing the percentage of the time that any method is within 5% of the maximum observed power among the remaining three quantitative methods (SUM-NR, ABSSUM-NR and MAXMEAN-NR). Results in Table 2 are for α = 0.05, but are similar for other α values (results not shown).

**Table 2:** Percentage of time method is within 5% of the maximum observed power

**Gene Set Size**	**Mean of log-ratios**	**Standard deviation of log-ratios**	**ABSSUM-NR**	**SUM-NR**	**MAXMEAN-NR**
2–5	0.02–0.24	< 1.0	72	33	15
		1.0+	98	12	16
	0.50–0.99	< 1.0	60	76	58
		1.0+	97	26	50
	1.0+	< 1.0	89	95	93
		1.0+	100	96	99
10–20	0.02–0.24	< 1.0	60	56	45
		1.0+	100	26	86
	0.50–0.99	< 1.0	67	95	93
		1.0+	100	75	87
	1.0+	< 1.0	99	99	100
		1.0+	100	100	100
50	0.02–0.24	< 1.0	57	78	69
		1.0+	100	66	99
	0.50–0.99	< 1.0	88	98	99
		1.0+	100	92	93
	1.0+	< 1.0	100	100	100
		1.0+	100	100	100

SUM-NR is typically optimal when the mean of the log-ratios is large, but the worst when the standard deviation is large. Further, ABSSUM-NR is typically best when the standard deviation of the log-ratios is large, as is the case for mixture distributions with a large mixing proportion, but performs poorly when the mean is large. MAXMEAN-NR is typically the second most optimal method of the three. These results correspond to arguments presented by Efron and Tibshirani [8] in which they argue that the MAXMEAN-NR statistic is the most robust to both shift (mean) and scale (standard deviation) changes. Efron and Tibshirani, however, only consider gene sets containing 20 genes. We find that the MAXMEAN-NR method is robust against both mean and standard deviation changes for gene sets of size 10, 20 and 50. However, MAXMEAN-NR performs poorly compared to ABSSUM-NR and SUM-NR when gene set sizes are small and the mean is small.

### Evaluating FET cutoffs

In the simulation study, we used four different cutoffs (3, 2, 1 and 1/2) for deciding whether or not a gene was "significantly regulated" based on the absolute value of its log-ratio. As we did when comparing quantitative methods, we consider only those cases where at least one of the four FET cutoffs gave adequate (> 80%) power. First, we found that FET with a cutoff of 2 or 3 never had the maximum observed power when compared to FET 1 or FET 1/2. We note, however, that our simulation study did not consider average set log-ratios above 2 which would likely be those situations where FET 2 or FET 3 may be optimal. When comparing FET 1 and FET 1/2 we found that, as a general rule, when at least a subgroup of genes in the set had an absolute mean log-ratio of expression values of at least 1.5, FET 1 provided the maximum observed power, and when the absolute mean log-ratios of expression values were less than 1.5, then FET 1/2 gave maximum observed power (details not shown).

### Comparing FET 1, FET 1/2 and MAXMEAN-NR

In Table 3 we compare FET 1, FET 1/2 and MAXMEAN-NR since FET 2 and 3, SUMSQ-NR and GSEA-NR were all shown to be less than optimal and ABSSUM-NR and SUM-NR were shown to be less robust than MAXMEAN-NR in most cases. To do this, we record, for different simulation settings, the percentage of the time that a method is within 5% of the maximum observed power (where the maximum observed power is the best power of the three methods considered here: FET 1, FET 1/2 and MAXMEAN-NR).

**Table 3:** Percentage of time method is within 5% of the maximum observed power

**Gene Set Size**	**Mean of log-ratios**	**Standard deviation of log-ratios**	**FET 1**	**FET 1/2**	**MAXMEAN-NR**
2–5	0.02–0.24	< 1.0	31	32	55
		1.0+	63	53	20
	0.50–0.99	< 1.0	25	19	83
		1.0+	66	70	71
	1.0+	< 1.0	58	66	99
		1.0+	100	76	99
10–20	0.02–0.24	< 1.0	20	51	69
		1.0+	100	100	86
	0.50–0.99	< 1.0	41	56	97
		1.0+	97	90	88
	1.0+	< 1.0	93	98	100
		1.0+	100	100	100
50	0.02–0.24	< 1.0	24	59	82
		1.0+	100	100	99
	0.50–0.99	< 1.0	62	87	99
		1.0+	100	100	93
	1.0+	< 1.0	100	100	100
		1.0+	100	100	100

Table 3 illustrates that MAXMEAN-NR performs well when compared to FET 1 or FET 1/2, often performing as well or better then either FET method. The few cases where FET methods performed better, tended to be with smaller means and larger standard deviations, cases when the ABSSUM-NR method was demonstrated to perform better than MAXMEAN-NR (see Table 2). When ABSSUM-NR was compared to FET 1/2 and 1 in the situations where MAXMEAN-NR does poorly, ABSSUM-NR was within 5% of maximum power in all cases [details not shown]. All results in Table 3, are for α = 0.05. While not shown here, results for other α values yielded similar results.

### Results of analyzing experimental data

As described in the *Methods: Experimental Data *section, we analyzed 18 different experimental comparisons taken from three different sets of experiments. Ten of the experimental comparisons were from *E. coli *and eight were from *S. typhimurium*. Table 4 presents the number of sets found as significantly regulated for each of the 9 gene set methods across four different α values for these 18 experiments.

**Table 4:** Number of sets found as significantly regulated across the 18 experiments

**Method**	**α = 0.05**	**α = 0.005**	**α = 0.0005**	**α = 0.0002**
FET 3	32	6	3	3
FET 2	78	23	11	11
FET 1	196	85	58	50
FET 1/2	338	109	54	45
GSEA-NR	522	137	61	50
SUMSQ-NR	311	64	34	31
ABSSUM-NR	444	124	66	57
SUM-NR	573	142	79	67
MAXMEAN-NR	613	181	91	74

In general, the findings from the experimental data were in line with findings from analysis of the simulated data. Briefly, 38 of the 141 sets (27%) found as significant only by the GSEA-NR method were sets with at least 10 genes, mean expression less than 0.10 and standard deviation less than 0.20, precisely the circumstances identified by simulation where GSEA-NR has increased false positives (see Table 1). Sets found as significant by SUMSQ-NR were virtually all found by ABSSUM-NR, but ABSSUM-NR found substantially more sets as significant (see Table 4). FET 2 and FET 3 found few sets as significant (see Table 4). Lastly, Table 4 illustrates that both SUM-NR and MAXMEAN-NR identify the most sets as significant at all α values.

### Biological significance of findings

In order to validate the statistical methods under study, the following section considers sets determined to be significantly regulated for consistency with what is known about the biology of the organisms. The emphasis of this analysis will be on those gene sets found to be significant by at least one of five methods considered valid based on the simulation and real data analyses presented earlier, namely FET 1, FET 1/2, ABSSUM-NR, SUM-NR, and MAXMEAN-NR. A complete listing of all significant sets for all five methods and all eighteen experiments is available in Additional File 1.

#### E. coli Acetyl Phosphate Mutants

Among the 8 different experimental comparisons that we considered for the two acetyl-phosphate mutants, only one was the focus of extensive analysis by Wolfe *et al*. [27] and, thus, is the only one considered here. In this comparison the *E. coli ackA *mutant (able to produce acetyl phosphate but not further metabolize it) is compared to the *E. coli pta-ackA *mutant (unable to produce acetyl phosphate). Our analysis shows that three gene sets (Bacterial Chemotaxis [19 genes], Flagellum [45 genes] and Ribosome LSU Bacterial [34 genes]) were significantly differentially expressed between the two mutants. The Flagellum gene set was identified by all five (FET 1, FET 1/2, ABSSUM-NR, SUM-NR, and MAXMEAN-NR) methods, the Bacterial Chemotaxis gene set was identified by all but FET 1 (p = 0.125) while the Ribosome LSU Bacterial set was identified only by SUM-NR and MAXMEAN-NR. These findings correspond well with the findings of Wolfe *et al*. [27] for genes that were significantly upregulated in the *pta-ackA *mutant versus the *ackA *mutant. The original study [27] also found genes associated with other processes significantly upregulated when comparing the *ackA *mutant to the *pta-ackA *mutant, however many of the genes found by Wolfe et al. are not part of the SEED [28] tool which was used for the creation of gene sets.

#### E. coli Sugar-acids

While the microarray data from the Sugar Acids experiments have not been analyzed in detail, a companion article has been published in which these data are used to corroborate specific findings on the regulation of the L-idonic acid pathway in *E. coli *[29]. Identification of the genes most highly induced when grown on L-idonate using a combination of standard techniques (LacZ fusions, RT-PCR, northern blotting) (see Table 6 in Bausch *et al*. [29]), implicates genes in the D-Gluconate and Ketogluconate [DGK] metabolism gene set. In line with these findings, our analysis of the microarray data comparing *E. coli *grown on idonate vs. glucose as well as a comparison of idonate plus gluconate vs. glucose also implicated the DGK gene set. The DGK set was found as significant in the idonate vs. glucose experiment by ABSSUM-NR, SUM-NR and MAXMEAN-NR but not by either FET 1 (p = 0.028) or FET 1/2 (p = 0.13), while the DGK set was found as significant by all methods except FET 1/2 (p = 0.0005) in the idonate plus gluconate vs. glucose experiment.

#### S. typhimurium experiments

Pathogenicity from *S. typhimurium *infection is conferred through the activity of genes encoded on a series of at least 5 gene clusters termed *Salmonella *pathogenicity islands (SPIs), though not all genes necessary for virulence are found on SPIs (for a review see Marcus *et al*. [30]). The growth conditions used in this study were designed to mimic different phases of *Salmonella *infection. In the following analysis, we will focus on the expression of SPIs in *hfq *and *smpB *mutants, which are global regulators of transcription and post-transcriptional processes in the cell [31,32]; mutant strains are attenuated for virulence *in vivo *[33,34].

For conditions mimicking an early infection stage [LB cultures], SPI-1, SPI-4 and SPI-5 are identified as significantly regulated in one or both mutants [*hfq *and *smpB*]. In the LB log cultures for the *hfq *mutant, SPI-1 is identified as significantly regulated relative to the wild-type by all five methods. SPI-1 is known to be involved in the intestinal phase/initial invasion of an infection [30], and *hfq *has recently been shown to affect the regulation of *hilA*, which is an important regulator of virulence genes encoded in SPI-1[34]. SPI-4 was identified by all methods except ABSSUM-NR (p = 0.00036), though this p-value could be considered borderline significant. SPI-1 and SPI-4 are co-regulated via *hilA *activity [35]. SPI-5 was identified as significantly regulated by ABSSUM-NR, SUM-NR and MAXMEAN-NR, but not by FET 1 (p = 0.024) or 1/2 (p = 0.073). SPI-5 encodes an effector protein (*sopB*) that is secreted by the type three secretion system (TTSS) of SPI-1 during invasion, and it has been shown that the *sopB *is co-regulated with SPI-1 via *hilA*[36]. While none of the methods identify SPI-5 as significant in the *smpB *mutant grown in LB log conditions, ABSSUM-NR, SUM-NR and MAXMEAN-NR calculate p-values very near the selected alpha of 0.0002 (p values ranging from 0.00028 to 0.00042). In contrast, FET 1 (p = 0.009) and FET 1/2 (p = 0.005) are not close to the selected alpha.

For conditions mimicking macrophage survival [MgM shock and dilution], SPI-2 is identified as significantly regulated in three of the four possible cases [shock and dilution for each of the two mutants] by FET 1, 1/2 and ABSSUM-NR, once by MAXMEAN-NR, but not at all by SUM-NR. SPI-2 is known to encode TTSS proteins, effector proteins, secretion system chaperones and transcriptional regulators involved in macrophage survival [30]. None of the five statistical methods of analysis identified SPI-3, which is also implicated in macrophage survival, as having significantly altered expression levels. This result suggests that *hfq *and *smpB *do not play a role in SPI-3 expression.

Many other non-SPI gene sets are implicated by one or more of the five statistical methods, however we do not offer a detailed analysis of these data here, merely noting that the results are in general agreement with what is known of *hfq *and *smpB *mutants in *Salmonella *and related bacteria (see Sittka *et al*. [34], Okan *et al*. [37] and references therein).

### Comparing simulation and real data analyses

Overall, the findings from simulation were similar to the findings from the real data analysis. Specifically, simulation and real data analysis both suggested that the GSEA-NR method may be overly sensitive to sets with few up or down regulated genes (false positives). Our finding that GSEA may be oversensitive is in line with other recent findings [4]. Dinu *et al*. [4] propose SAM-GS as an alternative to GSEA that doesn't have a false positive problem. We implement SAM-GS as SUMSQ-NR and consequently find that it never performs as well as ABSSUM-NR. Further, our simulation results corroborated results from Efron and Tibshirani [8] for replicated experiments. Specifically, ABSSUM-NR detects changes in standard deviation of log-ratios well, SUM-NR detects changes in mean log-ratios well, but MAXMEAN-NR is robust to detecting both types of changes.

When considering FET cutoff values, we find that 2 and 3 are not sensitive enough, picking up only a few significant sets in the real data analysis, while in the simulation FET 2 and 3 were never optimal. The simulation showed (Table 3) that the MAXMEAN-NR method was more powerful than either FET 1 or 1/2 in most cases, which was further corroborated in the real data analysis (Table 4) when MAXMEAN-NR identified many more sets as significant when compared to FET 1 or 1/2. In the few cases when MAXMEAN-NR was not more powerful than either FET 1 or 1/2 (small sample sizes), ABSSUM-NR performed well.

MAXMEAN-NR, SUM-NR and ABSSUM-NR all performed well when examining the significant sets for their biological relevance. Specifically, MAXMEAN-NR, SUM-NR and ABSSUM-NR were, in general, better than either FET 1 or 1/2 at consistently findings biologically relevant sets (see *Biological significance of findings*). In terms of biological significance, there was little difference which of the three non-cutoff based methods was used since each method failed to identify a biologically relevant set as significant at least two times. We note that in many cases when one of the three methods did not find a biologically relevant set at statistically significant using a strict α criteria, the set could be considered borderline significant.

## Discussion

### Non-cutoff based methods for prokaryotes

We have demonstrated how five methods for conducting gene set analysis can be implemented on experiments with few, if any, replicates, as is common for studies of prokaryotes. In line with previous results comparing cutoff [FET] to non-cutoff based methods, we find that cutoff based methods lack robustness and power. Further, we establish that the MAXMEAN-NR statistic is a powerful and robust statistic that correctly controls the type I error rate as long as sete sizes are at least 10. When set sizes are less than 10, ABSSUM-NR or SUM-NR may be a better choice.

GSEA-NR picked up a substantial number of false positives. Interestingly, the originally proposed GSEA procedure [2] [just a K-S test] suffered from the same problem, which was part of the reason for its updated (weighted) implementation [3]. That we found the same problems here suggests that either (1) any amount of weighting in the GSEA procedure is not enough to overcome the inherent false positive problems of GSEA (this suggestion is given by Dinu *et al*. [4]) or (2) that while the weights suggested for human data (the correlation between phenotype and genotype) are adequate, our use of the log-ratios of expression values as the weight in GSEA-NR, is not. Further research is necessary to answer this question.

SAM-GS [4] was developed to overcome the limitations of GSEA [2,3], but when implemented as SUMSQ-NR we found that it did not perform as well as ABSSUM-NR. ABSSUM-NR and SUMSQ-NR are relatively similar procedures. However, because SUMSQ-NR squares log-ratios, it sends log-ratios between 0 and 1 closer to 0, while increasing scores greater than 1. It is possible that this non-optimal handling of gene expression log-ratios between 0 and 1 (counting them less) contributes to its lack of power as compared to ABSSUM-NR.

In line with Efron and Tibshirani [8], we find that, in general, ABSSUM-NR provides the most power to detect changes in standard deviation, SUM-NR provides the most power to detect changes in mean and MAXMEAN-NR is robust to find both types of changes. This sets up an interesting practical problem. If gene sets are relatively consistently regulated, SUM-NR is likely the optimal choice of statistic since mean shifts would be all that would be expected. On the other hand, for gene sets in which subsets of genes are regulated, while others are not, ABSSUM-NR is the optimal choice, since the subset change will manifest itself, most noticeably, in a change in standard deviation. Since in today's environment we expect some gene sets to be consistently expressed, and others not, the MAXMEAN-NR statistic provides a robust approach to detecting both types of changes as significant.

### Choosing an α

While we considered a range of α values in the simulated data analysis, we only considered α = 0.0002 in the real data analysis. Alpha of 0.0002 was chosen because it was roughly equivalent to the Bonferroni adjusted alpha value of 0.05 divided by the number of gene sets, and was the smallest alpha value that could be reasonably considered for p-values based on 50,000 random gene sets. However, we demonstrated that biologically relevant sets may be found at p-values in the borderline significant region. This is not surprising since (1) the Bonferroni method is well-known to be overly conservative and (2) our null-hypothesis simulation study suggested that the false positive rate for most methods was much lower than the nominal rate. While not implemented here because it would hinder the comparison of different statistical methods, modern false positive control techniques like the False Discovery Rate should be implemented in practice.

### Limitations of gene sampling methods

All methods considered in this paper (FET, ABSSUM-NR, GSEA-NR, SUMSQ-NR, SUM-NR, and MAXMEAN-NR) are based on a gene-sampling model. Understanding gene sampling is crucial to understanding the limitations of these analyses. Gene sampling suffers from the major limitation that it always uses a competitive null hypothesis. That is, instead of having a null hypothesis of "This gene set is not regulated" the competitive null hypothesis says "This gene set is no more regulated than the other genes in the experiment which are not in the set." The implications of a competitive null hypothesis are that gene sets are competing with other genes outside of the gene set for significance. Thus, in experiments where many genes are regulated, gene sets with small, but real, mean log-ratios will be less likely to be detected as significant. Further, in experiments where very few genes are actually regulated there may be increased false positive findings. For a more in depth discussion of the implications of a competitive null hypothesis and gene sampling see Goeman and Buhlmann [20].

To date, all gene set analysis methods that do not rely on gene sampling (instead using subject sampling) require biological replicates because it is the replicates that are permuted to assess significance. Thus, for prokaryotic experiments with few biological replicates, gene sampling is necessary. There are, however, at least two options which should be investigated further for their utility in avoiding gene sampling for prokaryote microarray experiments. First, if microarray expression values and variability in typical experiments or under standard conditions could be quantified, it may be possible to characterize a general null hypothesis (gene set is not regulated) probability distribution. For a first step in this direction see Wren *et al*. [38]. Second, while multiple chips are often not used in prokaryotic microarray experiments, many prokaryote microarray chips have multiple probes for the same gene (technical replicates), which are then averaged to give the signal. Further work is necessary to evaluate and establish the utility of using information from multiple probes in gene set analyses.

### Limitations of the simulation study

While we implemented a relatively comprehensive simulation study, we must acknowledge some of its limitations. First, we did not examine true mean set log-ratios of more than 2. If true mean log-ratios more than 2 were examined, the likely result would be that using a cut off of 2 or 3 for FET would be more appealing than FET 1 or FET 1/2. However, as shown in both the analysis of real data and the simulation study, MAXMEAN-NR is robust to the problem of determining which FET cutoff is optimal, and would likely still appear optimal. Second, we modelled underlying gene set distributions as mixtures of normal distributions, a flexible modelling framework, which appeared to capture true variability well for the experimental data and gene sets we examined. However, further evaluation of the applicability of this model to other experiments and gene set definitions is necessary. Third, because all tests examined use a competitive null hypothesis, the simulated power of methods is directly related to the experimental distribution of log-ratios of expression values and so conclusions, while based on simulated data, are still somewhat dependent on the 18 experiments. To address this limitation we chose 18 experiments, from 2 different labs and a variety of experimental conditions. However, further experiments should be evaluated for their similarities/differences to data sets considered in this paper.

### Gene sets

In this paper we used gene sets as defined by SEED subsystems. Similarly, previous authors have used gene sets as defined by KEGG [39-41], the Gene Ontology [42], EcoCyc [43], and other data repositories. As was demonstrated earlier [see *Biological significance of findings: E. coli Acetyl Phosphate Mutants*], the gene set definitions are vitally important in determining the types of biological conclusions that can be made. Specifically, gene sets should be selected which are relevant and completely cover the possible biological systems under study.

## Conclusion

DNA microarrays provide the capability to measure genetic activity across the genome simultaneously as opposed to merely gene by gene, providing an understanding of genetic activity for prokaryotes at the organismal level. However, data analysis methods for this technology are still far from optimal. The findings discussed above represent a step in the direction of improved data interpretation for non or low-replicated DNA microarray experiments as are typical for prokaryotes.

Through simulation, real data analysis and evaluation of results for biological relevance, we find that MAXMEAN-NR offers a robust and powerful method of conducting gene set analysis on experiments with few or no replicates, though ABSSUM-NR or SUM-NR may be optimal for gene set sizes less than 10. Gene set analysis methods considered here are limited because they are gene sampling methods. Further work is needed to overcome the limitations of gene sampling methods for prokaryotes.

## Methods

### Extensions of gene set methods to non-replicated data via gene-sampling

We consider five non-cutoff based methods and FET (with four different cutoffs) in this paper. Each method is described briefly below. Significance for each of the non-cutoff based methods is determined using gene sampling (described in *Gene sampling and a competitive null hypothesis*).

#### GSEA-NR

GSEA was originally published by Mootha *et al*. [2], with a revised version proposed by Subramanian *et al*. [3]. GSEA has quickly become one of the most popular methods of conducting gene set analyses for replicated microarray experiments.

GSEA-NR (non-replicated sample version of GSEA) is implemented as follows. Let *L *be a list of genes sorted by their log-ratios of gene expression values. In GSEA-NR each gene in the set of interest is weighted according to the absolute value of its log-ratio. A weighted Kolmogorov-Smirnov statistic (termed the Enrichment Score) is computed, which finds the maximum deviation between the weighted empirical distribution function [EDF] of genes in the set of interest versus the unweighted EDF of genes not in the set. Practically speaking, the test statistic can be thought of as a non-parametric measure of the difference in the weighted distribution (e.g. histogram) of genes in the set versus the unweighted distribution of genes not in the set. In general, the mathematics of the method as proposed here is the same as the original method as presented in Subramanian *et al*. [3].

#### ABSSUM-NR

ABSSUM-NR finds the sum of the absolute values of the log-ratios of genes in the set of interest. ABSSUM-NR was recently considered by Efron and Tibshirani [8] and first proposed by [44].

#### SUMSQ-NR

SUMSQ-NR was recently proposed [4] as SAM-GS and is similar in nature to ABSSUM-NR. SUMSQ-NR finds the sum of the squared log-ratios of genes in the set of interest.

#### SUM-NR

SUM-NR simply finds the sum of the log-ratios of genes in the set of interest and was recently considered by Efron and Tibshirani [8].

#### MAXMEAN-NR

MAXMEAN-NR takes as the statistic the maximum of either (1) the average of the absolute value of the negative log-ratios times the percentage of genes in the set that have negative log-ratios or (2) the average of the positive log-ratios times the percentage of genes in the set that have positive log-ratios. MAXMEAN-NR was first considered by Efron and Tibshirani [8].

#### Fisher's exact test

Fisher's exact test is implemented by taking the sorted list of genes *L *and dichotomizing it by classifying some genes as up/down-regulated and others as not regulated. To implement these procedures we used four different cutoffs (3, 2, 1, and 1/2) for up/down-regulated based on the absolute values of log-ratios in *L*. FET then uses the hypergeometric distribution to find the p-value of the test. As described by Goeman and Buhlmann [20], Fisher's exact test methods are based on an implied gene sampling model.

### Gene sampling and a competitive null hypothesis

Gene sampling is a term coined by Goeman and Buhlmann [20] which means that the p-value of the gene set is based on random sets of genes. In other words, many, many gene sets of the same size as the gene set of interest are randomly selected, and the statistic of interest is computed on each of the random sets. The observed statistic is compared to the distribution of statistics from the random sets in order to obtain a p-value.

### Experimental Data

There are 3 sets of experiments under analysis. Two of these experiments involve experiments done on *E. coli *at the Oklahoma University Bioinformatics Core Facility by the Conway Lab [45]. The other experiment was conducted on *S. typhimurium *as a collaborative effort between faculty at the Oregon Health and Science University and the Sidney Kimmel Cancer Center. In the following sections we briefly outline the details of these experiments.

#### E. coli Acetyl Phosphate experiments

A series of six comparisons were made comparing gene expression values between two different knockout strains of *E. coli *K-12 (without *ackA *and without *ackA *and *pta*) plus the wild-type, grown in tryptone broth [TB] with or without acetate. The six comparisons analyzed in this paper correspond to the six comparisons available from the lab website [45] with much greater experimental detail given in [27]. The six comparisons are:

1- *ackA *mutant grown in TB plus 10 mM acetate vs. wildtype grown in TB

2- *ackA *mutant grown in TB vs. *ackA *mutant grown in TB plus 10 mM acetate

3- *ackA *mutant grown in TB vs. *pta *-*ackA *mutant grown in TB

4- *ackA *mutant grown in TB vs. wildtype grown in TB

5- *pta *-*ackA *mutant grown in TB vs. *ackA *mutant grown in TB plus 10 mM acetate

6- *pta*-*ackA *mutant grown in TB vs. wildtype grown in TB

#### E. coli Sugar Acids experiments

*E. coli *W1485 was grown on Neidhardt's MOPS minimal medium containing 0.2% sugar (glucose, gluconate, idonate, or mixtures) in 25 ml volumes in 250 ml Erlenmeyer flasks with gyrotary shaking at 300 rpm at 37°C. Cells were harvested for RNA extraction in mid-logarithmic growth phase (OD = 0.4) and total RNA was prepared and labeled, and Sigma-GenoSys Panorama membrane arrays were hybridized as described by [27,46]. There were four comparisons made in this set of experiments (all in log phase):

1- Gluconate vs. glucose

2- Idonate plus glucose vs glucose

3- Idonate plus gluconate vs. glucose

4- Idonate vs. glucose

Data is available at [45].

#### S. typhimurium experiments

*S. typhimurium *14028 cells were harvested (1), after growth at 30°C to log phase in LB (LBlog); (2), after growth at 30°C to stationary phase in LB (LBstat); (3) after transfer of a stationary phase culture grown in LB into magnesium-deficient MgM medium [26; MgM medium: 100 mM Tris-Cl, 5 mM KCl, 7.5 mM (NH4)2SO4, 0.5 mM K2SO4, 1 mM KH2PO4, 0.2% glycerol, 0.1% Casamino acids, 8 uM MgCl_2_] pH 5.0 and growth for four more hours at 30°C (MgMshock); (4) after 100-fold dilution of a stationary phase culture grown in LB into magnesium-deficient MgM medium pH 5.0 and growth at 30°C to log phase (MgM1:100). This procedure was performed on (A), wild type [WT] cells; (B), cells of an *hfq*^- ^(STM4361) knockout mutant; and (C), cells of an *smpB*^- ^(STM2688) knockout mutant. Eight total comparisons are considered in this paper: LBlog (*hfq vs*. WT; *smpB vs*. WT), LBstat (*hfq vs*. WT; *smpB vs*. WT), MgMshock (*hfq vs*. WT; *smpB vs*. WT), MgM1:100 (*hfq vs*. WT; *smpB vs*. WT).

Mutants were constructed using the Red-Swap method originally developed by Wanner and Datsenko [47] replacing the entire gene with a 24 nt gene-specific tag. Total bacterial RNA was isolated using the RNeasy kit (QIAGEN, Valencia, CA, USA). Superscript II reverse transcriptase (Invitrogen, Carlsbad, CA, USA) and random hexamers were employed during generation of fluorescently labeled cDNA. These targets were then hybridized onto custom-made non-redundant *Salmonella enterica *whole genome PCR product microarrays [48], using standard Corning GAPS slide protocols. Three independent biological replicates were interrogated for each condition and strain (36 hybridizations). Images were scanned using a Scanarray scanner (Perkin Elmer, Fremont, CA), and data extracted with ScanArrayExpress 3.0.1 and Quantarray 3.0 software packages. Differential gene expression was calculated with the WebArray online microarray data analysis platform [49], using Printtip LOESS and scale normalizations. The Salmonella data discussed in this publication have been deposited in NCBI's Gene Expression Omnibus [50] and are accessible through GEO Series accession number GSE11486 .

### Gene Sets from the SEED

The SEED is an open-source environment for genome annotation [28]. In it, genes are organized into subsystems, each representing a set of genes, often corresponding to a metabolic pathway. Based on gene sets defined by subsystems containing at least two genes from the SEED, there are 331 gene sets for *S. typhimurium *and 337 gene sets for *E coli*. Set sizes range from two to sixty-eight genes. Gene set definitions for *E. coli *and *S. typhimurium *are available as Additional files 2, 3, 4, 5.

### Simulation Study

For each gene in a gene set, the log-ratio of expression values is observed. We hypothesize that the resulting list of log-ratios for genes in a set is either normal or the mixture of two normal distributions. Unfortunately, since most gene sets have only a few genes, statistical tests to validate the normality or mixture of two normal distributions assumptions do not have sufficient power to detect practical deviations from these models [51].

Justification for the assumption of normality or mixture of normal distributions is as follows. First, if a gene set can be modelled by a normal distribution, this suggests that either (1) all genes in the gene set have the same true log-ratio of expression values and that deviations from the true log-ratio are observed due to technical and biological variability or (2) that most genes in a gene set have true log-ratios near to some number (*m*), and that fewer and fewer genes are in the set the farther you are from *m*. Option (1) provides a traditional "normal errors" assumption, assuming that a gene set contains genes that are very tightly regulated. Option (2) provides a reasonable model for some flexibility in the regulation of the genes in the set. If a gene set can be modelled by a mixture of two (or more) normal distributions, this can be thought of as a situation where a gene set is comprised of subsets of genes, where each subset of genes is normally distributed under option (1) or (2).

In order to explore the power of the various proposed methods for analyzing gene expression data in the context of gene sets, data was simulated from a variety of conditions. Table 5 shows the values of parameters chosen for the simulation.

**Table 5:** Simulation settings

**Simulation parameter**	**Levels**
π	1.0, 0.9, 0.8, 0.5
μ_1_	+/-2.0, +/- 1.0, +/- 0.5, +/- 0.25
μ_2_	+/- 1.5, +/- 0.5
σ_1_	0.25, 0.5
σ_2_	0.25, 0.5
Gene set sizes (n)	2, 5, 10, 20, 50

Specifically, *π *is the proportion of genes in the first normally distributed subset. If *π *= 1 then the gene set is normally distributed, and if *π *# 1 then the set of genes is a mixture of two normal distributions, where *πn *of the genes in the set are in the first normal distribution and *(1-π)n *are in the second. *μ*_*1 *_and *μ*_*2 *_are the means of the first and second subsets of genes, respectively. Similarly, *σ*_*1 *_and *σ*_*2 *_are the standard deviations of the first and second subsets of genes. The parameter settings were chosen to reflect the actual observed values of the mean, standard deviation, skewness and kurtosis of log-ratios of expression values within the experiments under study.

Seventeen combinations of *n *and π were run (*n *= 2 was not run for π = 0.95 and π = 0.80, and *n *= 5 was not run for π = 0.95). Further, there are 128 combinations of the other parameters (*μ*_*1*_, *μ*_*2*_, *σ*_*1*_, *σ*_*2*_; 8 × 4 × 2 × 2) for a total of 17 × 128 = 2176 different simulation settings. For each setting 2000 random sets were created. Each of the random sets was then used to compute the nine different gene set statistics (FET (3, 2, 1, and 1/2), GSEA-NR, SUMSQ-NR, ABSSUM-NR, SUM-NR and MAXMEAN-NR) for each of the 18 experiments under study. Each of these statistics was then compared to an empirical null distribution created by randomly sampling 50,000 sets of a given size, from each of the 18 experiments. Power was then computed as the percentage of the time that the null hypothesis was rejected out of the 2000 random sets for each of the 2176 simulation settings for four different α values (α = 0.05, 0.005, 0.0005, and 0.0002).

A small null hypothesis simulation was also conducted. For this simulation we chose settings of parameters that should reflect sets with little to no differential expression. In this simulation we used three different means: -0.05, 0, and 0.05, four different standard deviations: 0.01, 0.05, 0.10, and 0.20 and five set sizes (n = 2, 5, 10, 20, and 50), for a total of (3 × 4 × 5) 60 combinations. For each of the sixty combinations of simulation settings we generated 2000 random sets, and then ran each of the tests for each of the 18 experiments being studied.

## Authors' contributions

NT helped conceive of the study, extended the statistical methods, oversaw the simulations, and drafted much of the manuscript. AB helped conceive of the study, proposed refinements to statistical methods, oversaw the biological interpretation of data and drafted portions of the manuscript. MD helped conceive of the study, proposed refinements to statistical methods, extracted gene sets from the SEED, and refined preliminary versions of the manuscript. DV developed initial simulation routines and implemented final data analysis routines on the parallel computing cluster. FH conceived of the *S. typhimurium *study and participated in biological interpretation of the data. SP acquired the microarray data for *S. typhimurium*, assisted in technical interpretation of the microarray data, assisted in the creation of SPI gene sets, and deposited the data in GEO. RT assisted in initiating the project and preliminary data quality discussions. All authors read and approved the final manuscript.

## Supplementary Material

Additional file 1 **Significant gene sets for the 18 experiments**. A listing of the significant gene sets found by FET 1, FET 1/2, ABSSUM-NR, SUM-NR and MAXMEAN-NR for each of the 18 experiments, along with summary statistics for the distribution of log-ratios of expression values within each set. Click here for file

Additional file 2 **Gene set definitions for *E. coli***. This is a tab-delimited file with 4290 rows (one for each gene) and 338 columns. The first column gives the gene ID (b-number); the remaining 337 indicate whether (1) or not (0) the gene is in the gene set. There are 337 gene sets for *E. coli *based on SEED subsystems. Click here for file

Additional file 3 **Gene set names for *E. coli***. This is a tab-delimited file with 337 rows (one for each gene set) and 3 columns. The first column is the SEED subsystem name, the second is the numeric identifier for the set (1–337; corresponds to the order of sets in Additional file #2), and the third column indicates the number of genes in the set. Click here for file

Additional file 4 **Gene set definitions for *S. typhimurium***. This is a tab-delimited file with 4493 rows (one for each gene) and 332 columns. The first column gives the gene ID (STM number); the remaining 331 indicate whether (1) or not (0) the gene is in the gene set. There are 331 gene sets for *S. typhimurium *based on SEED subsystems. Click here for file

Additional file 5 **Gene set names for *S. typhimurium***. This is a tab-delimited file with 331 rows (one for each gene set) and 3 columns. The first column is the SEED subsystem name, the second is the numeric identifier for the set (1–331; corresponds to the order of sets in Additional file #4), and the third column indicates the number of genes in the set. Click here for file
