# Supplementary material for: Gene set analyses for interpreting microarray experiments on prokaryotic organisms
Source: BMC Bioinformatics. 2008 Nov 5;9:469. doi: 10.1186/1471-2105-9-469 (PMC2587482; doi:10.1186/1471-2105-9-469)
Supplement: Additional file 1 — Significant gene sets for the 18 experiments. A listing of the significant gene sets found by FET 1, FET 1/2, ABSSUM-NR, SUM-NR and MAXMEAN-NR for each of the 18 experiments, along with summary statistics for the distribution of log-ratios of expression values within each set. [file 1471-2105-9-469-S1.pdf]

Supplemental Table 1

Table of p-values of gene sets picked up as significant by FET 1, FET ½, ABSSUM-NR, SUM-NR or MAXMEAN-NR*

Note: p-values are bold and italicized if they are significant at the α=0.0002 level. Q1, Med and Q3 correspond to the 25th percentile, median and 75th percentile, respectively, of the log-ratios of gene expression scores of genes in that set and are meant as an indication of whether a set is up/down-regulated or a combination of both.

	Setsize	FET 1 (p-value)	FET ½ (p-value)	ABS-SUM-NR (p-value)	SUM-NR (p-value)	MAXMEAN-NR (p-value)	Q1	Med	Q3
**Acetyl Phosphate**									
**Expt 1**									
Serine-glyoxylate cycle	28	0.03208	0.00478	***0.00010***	0.00024	***0.00018***	0.03	0.30	0.82
Translation factors bacterial	24	0.00060	0.00366	***0.00006***	0.01324	0.00082	-0.01	0.44	0.83
Bacterial Cell Division	26	0.23717	***<0.00002***	0.00090	0.01528	0.00236	0.01	0.47	0.71
Bacterial Cytoskeleton	21	0.15259	***0.00018***	0.00096	0.04720	0.00520	0.00	0.43	0.77
F0F1-type ATP synthesis	9	0.00170	***0.00002***	***<0.00002***	***<0.00002***	***<0.00002***	0.78	0.99	1.44
Ribosome SSU Bacterial	22	***<0.00002***	***<0.00002***	***<0.00002***	***<0.00002***	***<0.00002***	0.60	0.92	1.11
Ribosome LSU Bacterial	34	***<0.00002***	***<0.00002***	***<0.00002***	***<0.00002***	***<0.00002***	0.16	0.89	1.16
Dyhydrogenase complexes	15	0.00022	***<0.00002***	***<0.00002***	***<0.00002***	***<0.00002***	0.51	0.82	1.70
TCA Cycle	22	***0.00004***	0.00033	***<0.00002***	***<0.00002***	***<0.00002***	0.09	0.56	1.83
Emben-Meyerhof and Gluconeogenesis	19	0.03121	0.00152	0.00152	***<0.00002***	***<0.00002***	0.22	0.58	0.85
General Secretion Pathway	15	0.63593	0.06042	0.22486	***0.00012***	0.01294	-0.62	-0.41	-0.01
Terminal cytochrome oxidases	10	0.02319	0.03247	0.00380	0.00084	***0.00012***	0.25	0.52	1.06
tRNA aminocylation	23	0.05823	0.00241	0.00352	0.00048	***0.00010***	0.19	0.47	0.85
Universal GTPases	16	0.00270	0.00114	0.00040	***<0.00002***	***<0.00002***	0.27	0.56	1.29

	Setsize	FET 1 (p-value)	FET ½ (p-value)	ABS-SUM-NR (p-value)	SUM-NR (p-value)	MAXMEAN-NR (p-value)	Q1	Med	Q3
**Acetyl Phosphate**									
**Expt 2**									
Lipoic acid metabolism	6	0.00759	0.00401	***0.00016***	0.00036	0.00056	-1.00	-0.93	-0.52
TCA Cycle	22	0.01372	0.00608	***0.00012***	0.00024	0.00040	-0.86	-0.32	-0.09
tRNA aminoacylation	23	0.41951	0.00042	***0.00008***	***0.00004***	***0.00006***	-0.79	-0.37	-0.16
Universal GPTases	16	0.00041	0.00308	***<0.00002***	***<0.00002***	***<0.00002***	-0.92	-0.40	-0.26
Glycine cleavage system	8	1.00000	***0.00013***	0.00048	0.0004	***0.00014***	-0.84	-0.64	-0.26
Dehydrogenase complexes	15	0.00031	***0.00003***	***<0.00002***	***<0.00002***	***<0.00002***	-1.01	-0.64	-0.39
NusA-TFII Cluster	4	***0.00015***	0.00894	***0.00008***	0.04784	0.0485	-1.53	-0.68	0.36
RNA polymerase bacterial	4	***0.00015***	0.00894	***0.00004***	***0.00012***	***0.00006***	-1.39	-1.24	-0.99
Translation factors bacterial	24	***<0.00002***	0.00064	***<0.00002***	***<0.00002***	***0.00002***	-1.14	-0.30	-0.06
F0F1-type-ATP-synthase	9	***<0.00002***	***<0.00002***	***<0.00002***	***<0.00002***	***<0.00002***	-1.25	-1.02	-0.81
Colanic Acid Biosynthesis	17	0.33083	0.69291	0.51186	***<0.00002***	0.30378	0.00	0.10	0.29
Ribosome SSU Bacterial	22	***<0.00002***	***<0.00002***	***<0.00002***	***<0.00002***	***<0.00002***	-1.29	-1.00	-0.87
Ribosome LSU Bacterial	34	***<0.00002***	***<0.00002***	***<0.00002***	***<0.00002***	***<0.00002***	-1.19	-0.99	-0.67
**Expt 3**									
Bacterial Chemotaxis	19	0.12507	***<0.00002***	***0.00016***	***<0.00002***	***<0.00002***	-0.70	-0.27	-0.10
Flagellum	45	***<0.00002***	***<0.00002***	***<0.00002***	***<0.00002***	***<0.00002***	-0.74	-0.35	0.00
Ribosome LSU Bacterial	34	1.00000	0.04811	0.01356	***<0.00002***	***0.00012***	-0.26	-0.15	-0.06
**Expt 4**									
Dehydrogenase complexes	15	0.02276	***0.00016***	0.00646	0.00184	***0.00014***	0.17	0.23	0.57
TCA Cycle	22	***0.00002***	0.00042	0.00106	***0.00004***	***0.00002***	0.07	0.24	0.83
Ribosome LSU Bacterial	34	0.42037	0.18754	0.01662	0.00020	0.00562	-0.39	-0.15	0.10

	Setsize	FET 1 (p-value)	FET ½ (p-value)	ABS-SUM-NR (p-value)	SUM-NR (p-value)	MAXMEAN-NR (p-value)	Q1	Med	Q3
**Acetyl Phosphate**									
**Expt 5**									
Bacterial Cell Division	26	0.02978	0.00096	***0.00018***	0.00584	0.00052	-0.69	-0.40	-0.16
Translation factors bacterial	24	0.00033	0.00042	***0.00002***	0.00092	***0.00012***	-0.85	-0.35	-0.13
Bacterial Cytoskeleton	21	0.10441	***0.00009***	0.00062	0.08624	0.01852	-0.69	-0.34	-0.03
Dehydrogenase complexes	15	0.33039	***<0.00002***	0.00024	***0.00008***	***0.00014***	-0.82	-0.62	-0.43
NusA-TFII Cluster	4	***0.00007***	0.01318	0.00116	0.24188	0.02556	-1.33	-0.68	0.28
RNA polymerase bacterial	4	***0.00007***	0.01318	0.00096	***0.00008***	***0.00004***	-1.26	-1.20	-0.99
Flagellum	45	***<0.00002***	0.00022	***<0.00002***	***<0.00002***	0.00040	0.00	0.24	0.72
F0F1-type ATP synthase	9	***0.00005***	***<0.00002***	***0.00002***	***<0.00002***	***<0.00002***	-1.32	-0.83	-0.64
Ribosome SSU bacterial	22	***<0.00002***	***<0.00002***	***<0.00002***	***<0.00002***	***<0.00002***	-1.04	-0.87	-0.74
Ribosome LSU bacterial	34	***<0.00002***	***<0.00002***	***<0.00002***	***<0.00002***	***<0.00002***	-1.06	-0.79	-0.50
Bacterial Chemotaxis	19	1.00000	0.16028	0.28602	***0.00004***	0.23144	0.02	0.19	0.49
Embden-Meyerhof and Gluconeogenesis	19	0.39846	0.01929	0.00624	0.00048	***0.00012***	-0.67	-0.36	-0.17
Universal GTPases	16	0.0649	0.02728	0.00138	0.00032	***0.00018***	-0.84	-0.40	-0.23

	Setsize	FET 1 (p-value)	FET ½ (p-value)	ABS-SUM-NR (p-value)	SUM-NR (p-value)	MAXMEAN-NR (p-value)	Q1	Med	Q3
**Acetyl Phosphate**									
**Expt 6**									
Dehydrogenase complexes	15	0.00073	0.00020	0.00262	***0.00004***	***<0.00002***	0.09	0.28	0.74
Protein chaperones	7	***0.00006***	0.02132	0.00398	0.00432	0.00036	0.12	0.28	1.28
Flagellum	45	***<0.00002***	***0.00015***	***<0.00002***	***<0.00002***	***<0.00002***	-0.02	0.21	0.56
TCA Cycle	22	***<0.00002***	***0.00008***	***0.00010***	***<0.00002***	***<0.00002***	0.01	0.10	1.20
**Sugar Acids**									
**Expt 1**									
Ribosome LSU Bacterial	34	0.00132	0.08022	***0.00008***	***0.00004***	***0.00002***	-0.24	0.11	0.28
D-Gluconate and Ketogluconate metabolism	19	0.18189	0.50054	0.06424	***0.00012***	0.00144	-0.18	-0.07	0.02
Leucine Biosynthesis	7	0.04050	0.01794	0.00090	0.00020	***0.00004***	-0.37	-0.04	-0.02
Translation factors bacterial	24	0.00111	0.00671	0.00392	0.00048	***0.00010***	-0.11	-0.01	0.15
**Expt 2**									
Translation factors bacterial	24	***0.00014***	0.00466	0.00052			-1.06	-0.61	-0.04
Ribosome LSU bacterial	34	***0.00004***	0.00480	***<0.00002***			-1.28	-0.46	0.23

	Setsize	FET 1 (p-value)	FET ½ (p-value)	ABS-SUM-NR (p-value)	SUM-NR (p-value)	MAXMEAN-NR (p-value)	Q1	Med	Q3
**Sugar Acids**									
**Expt 3**									
D-Gluconate and Ketogluconate metabolism	19	***0.00002***	0.00052	***<0.00002***	***<0.00002***	***<0.00002***	0.43	0.61	1.30
Branched-Chain Amino Acid Biosynthesis	16	0.18975	0.74433	0.24832	0.00004	0.02708	-0.53	-0.33	-0.21
Ribosome LSU Bacterial	34	0.08792	0.17889	0.02608	***<0.00002***	0.2958	-0.53	-0.22	0.18
**Expt 4**									
D-Gluconate and Ketogluconate metabolism	19	0.02818	0.12642	***<0.00002***	***<0.00002***	***<0.00002***	0.38	0.57	1.54
**Salmonella Experiments**									
**LB Log *hfq***									
Nitrate and nitrite ammonification	30	***0.00003***	***<0.00002***	***<0.00002***	***<0.00002***	***<0.00002***	0.29	0.60	0.91
Flagellum	44	***<0.00002***	***<0.00002***	***<0.00002***	***<0.00002***	***<0.00002***	-0.92	-0.60	-0.19
SPI 1	48	***<0.00002***	***<0.00002***	***<0.00002***	***<0.00002***	***<0.00002***	-1.11	-0.53	-0.24
SPI 4	6	***<0.00002***	***0.00019***	0.00036	0.00020	***0.00006***	-1.52	-1.14	-0.85
SPI 5	8	0.02418	0.07280	***0.00012***	***0.00004***	***0.00002***	-3.99	-1.54	-0.21
Propanedial Utilization	25	0.04202	0.01063	0.00290	***<0.00002***	***0.00006***	0.06	0.26	0.57

	Setsize	FET 1 (p-value)	FET ½ (p-value)	ABS-SUM-NR (p-value)	SUM-NR (p-value)	MAXMEAN-NR (p-value)	Q1	Med	Q3
**Salmonella Experiments**									
**LB Log *smpB***									
Threonine anaerobic catabolism	15	0.05296	0.33093	0.02794	***0.00016***	0.00350	-0.05	0.24	0.75
tRNA aminocylation	23	0.39153	0.1067	0.10576	***<0.00002***	0.00048	0.09	0.43	0.74
DNA structural proteins, bacterial	7	0.00576	0.01407	***0.00014***	***<0.00002***	0.00024	-1.87	-0.92	-0.40
Ribosome LSU Bacterial	35	***<0.00002***	***<0.00002***	***<0.00002***	0.05632	0.00050	-1.25	-0.15	0.78
Ribosome SSU Bacterial	22	***<0.00002***	***<0.00002***	***<0.00002***	0.00276	0.00236	-0.48	0.12	1.10
**LB stat *hfq***									
Phage shock protein (psp) operon	6	***<0.00002***	0.00285	***0.00018***	***<0.00002***	***<0.00002***	1.17	1.49	1.60
Conjugative Transfer	32	0.80206	0.28579	0.20766	***<0.00002***	***0.00016***	0.19	0.33	0.56
Ribosome LSU Bacterial	35	***<0.00002***	***<0.00002***	***<0.00002***	***<0.00002***	***<0.00002***	-1.48	-0.86	-0.52
Ribosome SSU Bacterial	22	***<0.00002***	***0.00004***	***<0.00002***	***<0.00002***	***<0.00002***	-1.35	-0.92	-0.30
Type IV secretion and conjugative transfer	33	0.81186	0.23043	0.25032	***<0.00002***	***0.00016***	0.18	0.32	0.55
SPI 2	44	0.00511	0.01173	0.00354	***<0.00002***	***<0.00002***	0.03	0.30	0.67
**LB Stat *smpB***									
Ribosome LSU Bacterial	35	0.01856	***0.00011***	0.00122	0.00492	0.00068	-0.64	-0.20	0.08
Phosphonate Metabolism	7	1.00000	0.30401	0.03650	***0.00012***	0.00248	0.30	0.37	0.66

	Setsize	FET 1 (p-value)	FET ½ (p-value)	ABS-SUM-NR (p-value)	SUM-NR (p-value)	MAXMEAN-NR (p-value)	Q1	Med	Q3
**Salmonella Experiments**									
**MgM1:100 *hfq***									
DNA structural proteins, bacterial	7	0.00039	0.06534	***0.00010***	***<0.00002***	***<0.00002***	-1.97	-1.09	-0.19
Ribosome SSU Bacterial	22	***<0.00002***	***0.00002***	***<0.00002***	***<0.00002***	***<0.00002***	-1.42	-1.11	-0.20
Ribosome LSU Bacterial	35	***<0.00002***	***<0.00002***	***<0.00002***	***<0.00002***	***<0.00002***	-1.80	-0.94	-0.13
SPI 2	44	***<0.00002***	***<0.00002***	***<0.00002***	0.10220	0.00118	-0.44	0.31	0.70
Conjugative Transfer	32	0.58375	0.06877	0.21252	***<0.00002***	0.00070	0.11	0.37	0.60
Type IV secretion and conjugative transfer	33	0.60074	0.08498	0.24770	***<0.00002***	0.00178	0.09	0.37	0.60
**MgM1:100 *smpB***									
Ribosome SSU Bacterial	22	***<0.00002***	***<0.00002***	***<0.00002***	***<0.00002***	***<0.00002***	0.06	0.84	1.29
Ribosome LSU Bacterial	35	***<0.00002***	***<0.00002***	***<0.00002***	0.00088	***<0.00002***	-0.77	0.45	1.42
SPI 2	44	***<0.00002***	***0.00003***	***<0.00002***	0.00084	***<0.00002***	-0.57	-0.07	0.43
**MgMshock *hfq***									
Ribosome SSU Bacterial	22	0.03235	***0.00015***	0.00430	0.00212	0.00030	-0.92	-0.44	-0.09
Propanedial utilization	25	***0.00011***	0.00069	0.00056	0.00620	0.00046	-0.90	-0.16	0.26
Transcription initiation bacterial sigma factors	9	***0.00012***	***0.00018***	0.00660	0.01436	0.00708	-0.06	0.53	0.90
Phage shock protein (psp) operon	6	0.17876	0.00106	0.01026	***<0.00002***	0.00166	0.57	0.73	0.90

	Setsize	FET 1 (p-value)	FET ½ (p-value)	ABS-SUM-NR (p-value)	SUM-NR (p-value)	MAXMEAN-NR (p-value)	Q1	Med	Q3
**Salmonella Experiments**									
**MgM shock *smpB***									
Ribosome SSU Bacterial	22	***0.00005***	0.00251	0.00106	***0.00004***	***<0.00002***	-1.20	-0.48	-0.04
SPI 2	44	***<0.00002***	***<0.00002***	***<0.00002***	0.09992	0.00814	-0.37	0.10	0.49

*Because of its large overlap with other gene sets, significance for the EGS prediction gene set is not included in this table.

E coli Acetyl Phosphate Experiments

1. ACP_ACK_AC_V_WT_LO---ackA plus added acetate vs. wildtype
2. ACP_ACK_V_ACK_AC_LO---ackA mutant vs ackA plus added acetate
3. ACP_ACK_V_PTAACK_LO---ackA mutant vs. pta-ackA mutant
4. ACP_ACK_V_WT_LO--ackA mutant vs. wildtype
5. ACP_PTA_V_ACK_AC_LO---pta ackA mutant vs. ackA mutant plus added acetate
6. ACP_PTA_V_WT_LO---pta ackA mutant vs. wildtype

E coli Sugar Acids experiments

1. GNT_V_GLC----minimal gluconate vs. glucose in log phase
2. IDN_GLC_V_GLC---minimal idonate plus glucose vs. glucose in log phase
3. IDN_GNT_V_GLC---minimal idonate plus gluconate vs. glucose in log phase
4. IDN_V_GLC---minimal idonate vs. glucose in log phase
